# 2545: Integration of HMIS and UI Health Cerner Clinical System to enable data sharing about homeless individuals

**DOI:** 10.1017/cts.2017.88

**Published:** 2018-05-10

**Authors:** Neil Bahroos, Subhash Kumar Kolar Rajanna, Stephen B. Brown, Padma Thangaraj, David Melnick, Angela Freeman

**Affiliations:** Center for Clinical and Translational Science, University of Illinois at Chicago, Chicago, IL, USA

## Abstract

OBJECTIVES/SPECIFIC AIMS: This research project envisions the integration of Homeless Management Information System (HMIS) and UI Health Cerner electronic medical record (EMR) system with the following goals: (1) enable sharing of data about the status of the housing insecure and homeless. (2) Identify and match patient record accurately. (3) Record housing insecurity or homelessness information with structured data elements in the EMR. METHODS/STUDY POPULATION: We created a Master Person Index (MPI) of the homeless individuals from HMSI using OpenEMPI software package, which is an open source implementation of an Enterprise Master Patient Index (EMPI). An entity model was generated based on the selective data elements from HMIS database, which were relevant for the patient identity management and healthcare service management. An automated script was implemented to extract data from HMIS and load it into OpenEMPI to build the MPI. Once the MPI is setup, the Emergency Department users were able to perform patient identity matching and confirm housing insecure or homeless status of their patients by querying the index using the web-based tool. We developed structured data elements to record homelessness information, which will allow us to measure the prevalence of this risk among patients. We are also exploring the possibility to integrate the systems the using the IHE PIX/PDQ profile, which provides ways for healthcare applications to query a patient information server for a patient based on user-defined search criteria, and retrieve a patient’s information directly into the application. RESULTS/ANTICIPATED RESULTS: We implemented a MPI of homeless individuals, which would allow the emergency department users to perform patient identity matching of housing insecure or homeless patients, without undue privacy intrusions. We are confident that IHE PIX/PDQ profile is able to support the integration of healthcare and housing and homeless services systems and enable the data sharing in an efficient way. DISCUSSION/SIGNIFICANCE OF IMPACT: The project addressed the gap in the sharing of data about housing insecure or homeless persons between healthcare and housing and social services that will result in improvements in coordination of care, reduce the cycle time from recognition of risk to the referral to housing and services and improve health outcomes and residential stability. Successful completion of this integration project will give us a model that we can scale to many other communities.

